# Novel Assays of Thrombogenic Pathogenicity in the Antiphospholipid Syndrome Based on the Detection of Molecular Oxidative Modification of the Major Autoantigen β_2_-Glycoprotein I

**DOI:** 10.1002/art.30383

**Published:** 2011-09

**Authors:** Yiannis Ioannou, Jing-Yun Zhang, Miao Qi, Lu Gao, Jian Cheng Qi, De-Min Yu, Herman Lau, Allan D Sturgess, Panayiotis G Vlachoyiannopoulos, Haralampos M Moutsopoulos, Anisur Rahman, Charis Pericleous, Tatsuya Atsumi, Takao Koike, Stephane Heritier, Bill Giannakopoulos, Steven A Krilis

**Affiliations:** 1 St. George Hospital and University of New South WalesSydney, New South Wales, Australia; 2 St. George Hospital and University of New South WalesSydney, New South Wales, Australia; 3 St. George Hospital and University of New South WalesSydney, New South Wales, Australia; 4 Metabolic Disease Hospital and Tianjin Medical UniversityTianjin, China; 5 National University of Athens Medical SchoolAthens, Greece; 6 University College LondonLondon, UK; 7 Hokkaido University School of MedicineSapporo, Japan; 8 The George Institute for Global Health and Sydney UniversitySydney, New South Wales, Australia

## Abstract

**Objective:**

Beta-2-glycoprotein I (β_2_GPI) constitutes the major autoantigen in the antiphospholipid syndrome (APS), a common acquired cause of arterial and venous thrombosis. We recently described the novel observation that β_2_GPI may exist in healthy individuals in a free thiol (biochemically reduced) form. The present study was undertaken to quantify the levels of total, reduced, and posttranslationally modified oxidized β_2_GPI in APS patients compared to various control groups.

**Methods:**

In a retrospective multicenter analysis, the proportion of β_2_GPI with free thiols in serum from healthy volunteers was quantified. Assays for measurement of reduced as well as total circulating β_2_GPI were developed and tested in the following groups: APS (with thrombosis) (n = 139), autoimmune disease with or without persistent antiphospholipid antibodies (aPL) but without APS (n = 188), vascular thrombosis without APS or aPL (n = 38), and healthy volunteers (n = 91).

**Results:**

Total β_2_GPI was significantly elevated in patients with APS (median 216.2 μg/ml [interquartile range 173.3–263.8]) as compared to healthy subjects (median 178.4 μg/ml [interquartile range 149.4–227.5] [*P* < 0.0002]) or control patients with autoimmune disease or vascular thrombosis (both *P* < 0.0001). The proportion of total β_2_GPI in an oxidized form (i.e., lacking free thiols) was significantly greater in the APS group than in each of the 3 control groups (all *P* < 0.0001).

**Conclusion:**

This large retrospective multicenter study shows that posttranslational modification of β_2_GPI via thiol-exchange reactions is a highly specific phenomenon in the setting of APS thrombosis. Quantification of posttranslational modifications of β_2_GPI in conjunction with standard laboratory tests for APS may offer the potential to more accurately predict the risk of occurrence of a thrombotic event in the setting of APS.

The antiphospholipid syndrome (APS) is an autoimmune condition characterized by vascular thrombosis of the arterial and/or venous systems as well as recurrent miscarriages (1). Beta-2-glycoprotein I (β_2_GPI) is the major autoantigen in APS (2). A number of studies have provided robust evidence that autoantibodies to β_2_GPI are a significant risk factor for arterial thrombosis in young adults (3, 4). In vivo and ex vivo studies by multiple groups have shown anti-β_2_GPI autoantibodies to be directly thrombogenic (5).

At present it is not possible to stratify the risk for development of thrombosis in antiphospholipid antibody (aPL)–positive patients based on clinical features or use of currently available laboratory assays (6). The development of novel assays that could be used to stratify future thrombosis risk in patients with APS would hold immense clinical utility in informing the decision as to whether initiation of prophylactic therapy or intensification of therapy is warranted.

Beta-2-glycoprotein I is an evolutionarily conserved 50-kd protein circulating in the blood in relative abundance (∼4 μ*M*) (7). The physiologic role of β_2_GPI is pleiotropic, with functional studies implicating a role in processes relating to coagulation (8), angiogenesis (9), and clearance of apoptotic cells (10). The crystal structure of β_2_GPI, which has been ascertained based on the purified native protein, reveals that it does not possess free thiols (11, 12). We have recently shown, however, that in vivo β_2_GPI circulates in a free thiol form and that this free thiol form of β_2_GPI is involved in the protection of endothelial cells against oxidative stress–induced cell injury (13). Beta-2-glycoprotein I can also participate in redox thiol-exchange reactions by acting as a substrate for oxidoreductase enzymes such as thioredoxin 1 (14). However, the proportion of β_2_GPI circulating in the reduced state is unknown. Also unknown is whether the redox state of this autoantigen differs in patients with pathogenic anti-β_2_GPI antibodies and a history of thrombosis.

In the present study we demonstrated that, in serum/plasma derived from healthy subjects, β_2_GPI exists in a reduced biochemical state as the dominant molecular phenotype. Detailed in vitro quantitative assays to assess the levels of total and reduced β_2_GPI were developed and used to screen >450 samples. Levels of both total and oxidized β_2_GPI were found to be elevated in patients with APS as compared to disease and healthy control groups. These findings have implications with respect to understanding the antigenic drive for pathogenic aPL, as well as the potential for development of assays for purposes of thrombosis risk stratification.

## PATIENTS AND METHODS

### Patient samples

Samples were collected through an international collaborative multicenter effort involving 5 centers (University of New South Wales [Sydney, Australia], University of Athens [Athens, Greece], University College London [London, UK], Tianjin Medical University [Tianjin, China], and Hokkaido University School of Medicine [Sapporo, Japan]). An APS group, 2 disease control groups, and 1 healthy control group were studied. The disease control groups consisted of an autoimmune disease group (with or without aPL, but with no clinical features of APS) and a clinical event control group (clinical features of APS, but no aPL or autoimmune disease).

#### APS group

A total of 139 samples from patients with APS were collected and analyzed (24 from Sydney, 38 from Athens, 22 from London, and 55 from Sapporo). Every APS patient fulfilled the revised consensus classification criteria for vascular thrombosis–associated APS (1). All serologic tests for aPL were performed using standard commercially available kits and in accordance with the revised classification criteria. A venous thrombotic event was diagnosed based on a combination of clinical assessment and appropriate imaging with either Doppler ultrasonography or venography to confirm deep venous thrombosis, or isotope ventilation/perfusion scanning or computed tomography (CT) (with or without angiography) to confirm pulmonary embolism. An arterial event was diagnosed based on clinical findings along with one or more of the following: electrocardiographic evidence of myocardial ischemia or infarction, confirmation of infarction by brain CT or magnetic resonance imaging, or confirmation of peripheral vascular disease or arterial thrombosis by Doppler ultrasonography or angiography.

#### Autoimmune disease control group

Of the 189 autoimmune disease controls, samples from 188 were analyzed (42 from Sydney, 43 from Athens, 29 from London, and 74 from Sapporo). One sample (from a patient with systemic lupus erythematosus [SLE] and no aPL) was found to be deficient in β_2_GPI and was withdrawn from the study. Among the autoimmune disease controls, 74 had persistently positive serologic findings for aPL satisfying the serologic component of the APS classification criteria (1), but did not have APS given the lack of a clinical event. All patients with SLE fulfilled the American College of Rheumatology revised classification criteria (15), and those with Sjögren's syndrome fulfilled the revised European classification criteria (16).

#### Clinical event control group

Thirty-eight samples from aPL-negative patients with a clinical event were collected and analyzed (26 from Sydney and 12 from Tianjin). Clinical events were diagnosed as described above for the APS group.

#### Healthy control group

Samples from 93 healthy controls were collected, 92 of which were analyzed (28 from Sydney, 35 from Athens, and 29 from Sapporo). One healthy control sample was found to be deficient in β_2_GPI by standard enzyme-linked immunosorbent assay (ELISA) and was withdrawn from the study.

Demographic and clinical details of the study groups are summarized in Table 1. Institutional ethics approval for patient sampling was attained from each center participating in the study, and informed consent was obtained from all subjects prior to venipuncture. Assays were performed under blinded conditions with regard to the underlying diagnosis.

**Table 1:** Demographic and clinical characteristics of the groups studied*

		Control groups		
		
	APS	Autoimmune disease	Clinical event	Healthy
Patients	139	188	38	92†
Female	111 (79.9)	164 (87.2)	21 (55.3)	58 (63.0)
Age, median years	43	42	55.5	35
Race				
Caucasian	82	110	26	56
Asian	56	77	12	36
Afro-Caribbean	1	1	0	0
Autoimmune disease				
Total	75 (54.0)	188 (100)	1 (2.6)	0 (0)
SLE	58 (41.7)	106 (56.4)	1 (2.6)	–
SS	8 (5.8)	30 (16.0)	1 (2.6)	–
Other	10 (7.2)	58 (30.9)	–	–
Thrombosis				
Total	139 (100)	0 (0)	38 (100)	0 (0)
Arterial	80 (57.6)	–	21 (55.3)	–
Venous	72 (51.8)	–	20 (52.6)	–
aPL positive				
Total	139 (100)	74 (39.4)	0 (0)	0 (0)
aCL	93 (66.9)	43 (22.9)	0 (0)	–
Anti-β_2_GPI	79 (56.8)	29 (15.4)	0 (0)	–
LAC	89 (64.0)	47 (25.0)	0 (0)	–
Antithrombotic therapy				
Total	103 (74.1)	54 (28.7)	29 (76.3)	0 (0)
Anticoagulant	58 (41.7)	52 (27.7)	6 (15.8)	–
Antiplatelet	63 (45.3)	3 (1.6)	23 (60.5)	–

* Except where indicated otherwise, values are the number (%). APS = antiphospholipid syndrome; SLE = systemic lupus erythematosus; SS = Sjögren's syndrome; aPL = antiphospholipid antibody; aCL = anticardiolipin antibody; LAC = lupus anticoagulant.

† One sample from this group was subsequently withdrawn from analysis because standard enzyme-linked immunosorbent assay revealed it to be deficient in β_2_-glycoprotein I (β_2_GPI).

### Chemicals and reagents

HEPES and streptavidin beads were purchased from Sigma. *N*-(3-maleimidylpropionyl) biocytin (MPB) was purchased from Invitrogen. All other chemicals were of reagent grade.

### Proteins

Bovine serum albumin (BSA), alkaline phosphatase (AP)–conjugated anti-mouse IgG, AP-conjugated anti-rabbit IgG, and AP-conjugated anti-human IgG were from Sigma. Purified native human β_2_GPI was from Haematologic Technologies and also sourced as a kind gift from Dr. Inger Schousboe (University of Copenhagen, Denmark). Affinity-purified murine IgG2 anti-β_2_GPI monoclonal antibody (mAb) 4B2E7 (previously designated “mAb number 16”) and affinity-purified rabbit anti-β_2_GPI polyclonal antibody were produced as previously described (17, 18). Isotype control rabbit polyclonal IgG was purchased from BD PharMingen.

### Assay for quantifying the absolute proportion of serum β_2_GPI that can be labeled with MPB

With the demonstration that β_2_GPI exists in vivo in a reduced state with free thiols (13), it was then pertinent to determine the absolute proportion of total β_2_GPI that circulates in this reduced state. This was done in experiments with a sample of pooled serum derived from 10 healthy volunteers. The sex and age distribution of the pooled serum sample was chosen to match the APS disease group.

MPB-labeled and non–MPB-labeled serum samples were acetone precipitated to remove free MPB as described previously (13). The protein pellets were then dissolved in phosphate buffered saline (PBS)–0.1% Tween to a final dilution of 4,000-fold (total volume 1,400 μl), and streptavidin beads (50 μl) were added. After incubation with streptavidin beads (1 hour at 4°C), the beads were removed by centrifugation for 2 minutes at 3,000*g* and the supernatants assayed for β_2_GPI. The proportion of β_2_GPI that was labeled with MPB was calculated as (optical density at 405 nm [OD_405_] of the biotin-depleted MPB-labeled sample/OD_405_ of the biotin-depleted non–MPB-labeled sample) × 100. Validation of this method is described in full in the supplementary information (available in the online version of this article at http://onlinelibrary.wiley.com/journal/10.1002/(ISSN)1529-0131).

### Assay for quantifying total human β_2_GPI

A sandwich ELISA for quantifying total β_2_GPI levels within serum/plasma samples was performed based on a previously published method (19), with modifications. Briefly, a high-binding 96-well plate was coated overnight at 4°C with rabbit polyclonal anti-human β_2_GPI (10 n*M*/well). Plates were washed 4 times with PBS–0.1% Tween and then blocked with 2% BSA/PBS–0.1% Tween for 1 hour at room temperature. Following washing, 100 μl of anti-human β_2_GPI mouse mAb (clone 4B2E7) was added (10 n*M*/well, diluted in 0.25% BSA/PBS–0.1% Tween) and then 100 μl of the patient sample diluted 4,000-fold in PBS–0.1% Tween was coincubated for 1 hour at room temperature. After washing 4 times with PBS–0.1% Tween, AP-conjugated goat anti-mouse IgG was added (1:1,500 dilution) and incubated for 1 hour at room temperature, and samples read at OD_405_ after addition of chromogenic substrate. An in-house standard, consisting of pooled serum from 10 healthy controls, was used to construct a standard curve for every ELISA. The level of β_2_GPI in the pooled-serum in-house standard was determined initially using a β_2_GPI in-house standard curve and then validated with a calibrator from a commercially available β_2_GPI quantification kit (Hyphen BioMed). Each new batch of the pooled-serum in-house standard was recalibrated against the commercial calibrator. Samples were assayed in duplicate.

Within-plate coefficients of variation (CVs) for this ELISA were calculated by running 10 duplicates of the same patient sample on a single plate. Between-plate CVs were calculated by taking 10 independent assays performed consecutively on separate days and calculating the CV based on the variation of the number obtained by dividing the OD of the standard at 4,000-fold dilution by the OD of the standard at 8,000-fold dilution for each plate.

### Assay for measuring the relative amount of β_2_GPI with free thiols within patient samples as compared to a pooled-serum in-house standard sample

The amount of β_2_GPI with free thiols in patient samples relative to the standard sample was assayed as previously described (13), with minor modifications. Measurement of the amount of β_2_GPI that is reduced is based on labeling of free thiols of β_2_GPI with the biotin-conjugated selective free thiol binding reagent MPB, capturing biotin-labeled proteins on a streptavidin plate, and detecting the presence of MPB-labeled β_2_GPI with a specific anti-β_2_GPI mAb. The mean ± SD within-plate CV for this ELISA is 5.08 ± 3.09%, and the between-plate CV is 6.25% (13).

MPB (4 m*M*) was added to 50 μl of patient plasma or serum and incubated for 30 minutes at room temperature in the dark with agitation, diluted 50-fold in 20 m*M* HEPES buffer (pH 7.4), and incubated for a further 10 minutes at room temperature in the dark. Unbound MPB was then removed by acetone precipitation. The protein pellet was resuspended in PBS–0.05% Tween (final dilution 100-fold). The samples were then diluted a further 40-fold (4,000 times final), added in duplicate to a streptavidin-coated 96-well plate (100 μl/well; Nunc), and incubated for 90 minutes at room temperature. Prior to addition of MPB-labeled serum samples, streptavidin-coated plates were washed 3 times with PBS–0.1% Tween and blocked with 2% BSA/PBS–0.1% Tween. After washing 3 times with PBS–0.1% Tween, the murine anti-β_2_GPI mAb (clone 4B2E7) was added (25 n*M*) and incubated for 1 hour at room temperature. After 3 further washings with PBS–0.1% Tween, AP-conjugated goat anti-mouse IgG (1:1,500 dilution) was added for 1 hour at room temperature and samples read at 405 nm after addition of chromogenic substrate. For each experiment, the pooled in-house standard used for the above-described β_2_GPI quantification ELISA was MPB labeled, acetone precipitated, and used as an internal control and standard. The degree of MPB labeling in each patient sample was expressed as a percentage of that observed with the pooled in-house standard, after correction for the total amount of β_2_GPI. The proportion of non–MPB-labeled β_2_GPI represents the oxidized form of the molecule.

### Statistical analysis

Box plots were created to depict the distributions of β_2_GPI across groups. Medians and interquartile ranges (IQRs) were calculated. For comparisons between individual samples, the Mann-Whitney U test was used. Odds-ratios (ORs) and 95% confidence intervals (95% CIs) of exposure or disease incidence were computed using logistic regression. Adjustment for age and sex was carried out to remove potential confounders linked to these predictors.

## RESULTS

### A significant proportion of β_2_GPI in vivo in healthy volunteers circulates in the reduced form

**Figure 1:**
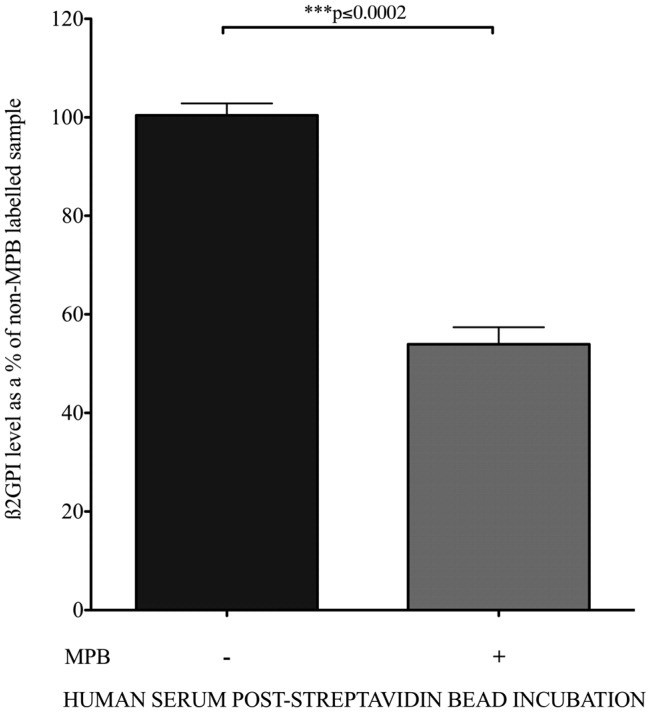
Beta-2-glycoprotein I (β_2_GPI) with free thiols represents a large proportion of total circulating β_2_GPI in vivo. Pooled serum from 10 healthy volunteers was labeled with *N*-(3-maleimidylpropionyl) biocytin (MPB) (4 m*M*) or treated with control buffer alone, after which the MPB-labeled proteins were depleted by incubation with streptavidin beads. Both samples were then centrifuged at 3,000*g* for 10 minutes to remove the beads, and an enzyme-linked immunosorbent assay for total β_2_GPI was performed on the supernatant of both MPB-labeled and non–MPB-labeled samples post–streptavidin incubation. The relative reduction (in optical density) of the MPB-labeled sample as compared to the non–MPB-labeled sample indicates the relative amount of β_2_GPI with free thiols labeled with MPB. Values are the mean ± SD.

We have recently demonstrated that β_2_GPI circulates in vivo in a reduced form (13), and we therefore wished to determine the absolute proportion of β_2_GPI that is in this biochemically reduced state. This was investigated using a sample of human serum pooled from 10 healthy volunteers. Figure 1 shows that a mean of 45.6% of β_2_GPI in pooled serum from healthy subjects was labeled with the biotin-conjugated free thiol binding reagent MPB. Validation of this method is demonstrated in detail in Supplementary Figure 1, available in the online version of this article at http://onlinelibrary.wiley.com/journal/10.1002/(ISSN)1529-0131).

### Total β_2_GPI levels are elevated in APS and are associated with thrombogenic pathogenicity in aPL-positive patients

Given that biochemically reduced β_2_GPI was found to represent a large proportion of circulating β_2_GPI in healthy subjects, it was then relevant to ascertain whether this level was altered in patients with APS as compared to both disease control and healthy control groups. Serum or plasma levels of total β_2_GPI were quantified in each individual patient sample so that a relative proportion of reduced and oxidized β_2_GPI could be calculated for each sample.

The assay used for detecting total levels of β_2_GPI in patient serum and plasma was optimized for use with in-house anti-β_2_GPI antibodies, as shown in Supplementary Figure 2 (http://onlinelibrary.wiley.com/journal/10.1002/(ISSN)1529-0131). The within-plate CV for this assay was 5.8% and the between-plate CV was 3.3%, indicating good reproducibility.

**Figure 2:**
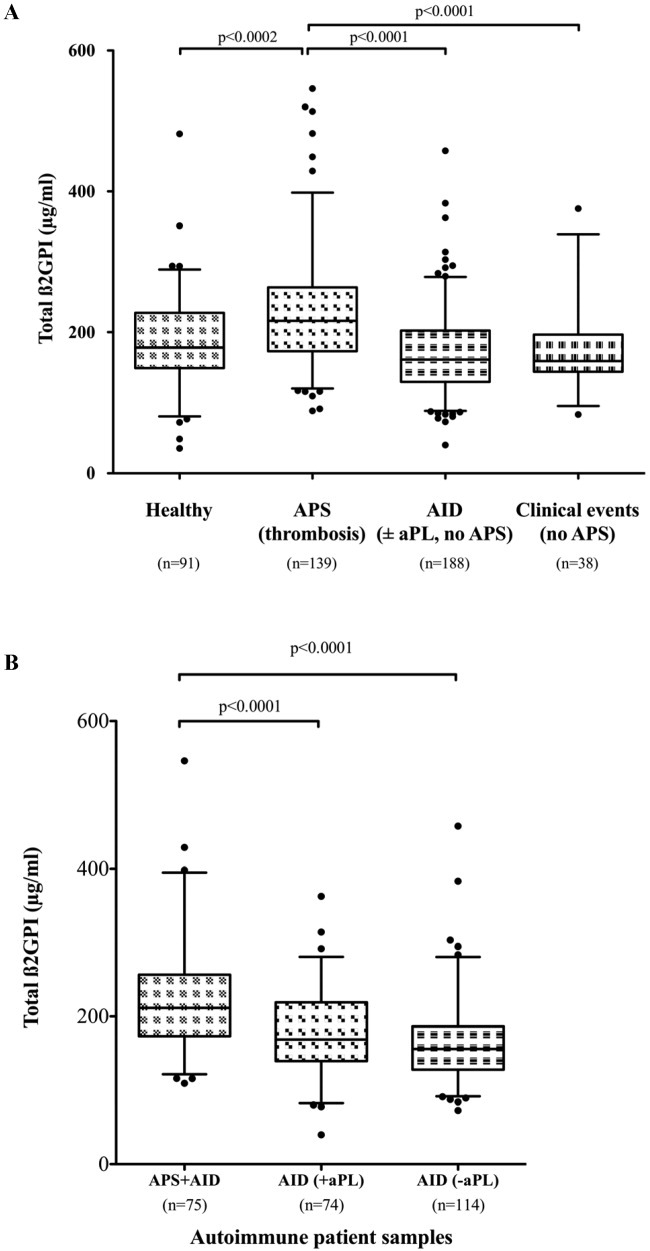
Elevated levels of β_2_-glycoprotein I (β_2_GPI) in patients with the antiphospholipid syndrome (APS). **A**, Total β_2_GPI in the serum of patients with thrombosis-associated APS and in the serum of patients in the 3 control groups, i.e., healthy controls, patients with autoimmune disease (AID) with or without antiphospholipid antibodies (aPL) but without APS, and patients with clinical thrombotic events without APS. **B**, Total β_2_GPI in the serum of patients in the APS group who had an autoimmune disease compared to patients in the autoimmune disease control group who were positive for aPL and patients in the autoimmune disease control group who were negative for aPL. Elevated levels of β_2_GPI were demonstrated only when aPL positivity was combined with a thrombotic clinical event. Data are presented as box plots, where the boxes represent the 25th to 75th percentiles, the lines within the boxes represent the median, and the lines outside the boxes represent the 10th and 90th percentiles. Circles indicate outliers.

The median level of total β_2_GPI in the healthy control group was 178.4 μg/ml (IQR 149.4–227.5) (n = 91). In addition to healthy controls, an autoimmune disease control group (autoimmune disease with or without aPL but without APS) and a clinical event control group (thrombosis without aPL) were included, as described above. As shown in Figure 2A, the concentration of total β_2_GPI was significantly higher in the APS group (median 216.2 μg/ml [IQR 173.3–263.8]) (n = 139) as compared to the healthy control group (*P* < 0.0002), the autoimmune disease control group (*P* < 0.0001), and the clinical event control group (*P* < 0.0001). Compared to healthy controls, cases were twice as likely to have an elevated β_2_GPI level (defined as plasma levels ≥200 μg/ml). The effect remained after adjustment for age and sex (OR 2.2 [95% CI 1.2–3.9]). Given that the odds ratios of disease and of exposure can be considered the same, this translates to a 2-fold increase in thrombosis for patients with elevated β_2_GPI levels, in the absence of further confounding effects. The association was stronger when the comparison was with the control group consisting of patients with autoimmune disease with or without aPL (OR 4.6 [95% CI 2.9–7.5]). It is also possible to treat total β_2_GPI as a continuous variable in the model. When this was done, the results were consistent with the other findings (i.e., there was a strong positive association between total β_2_GPI level and thrombosis risk).

Figure 2B shows that elevated β_2_GPI levels were observed only when persistent aPL positivity was combined with a thrombotic event, thus fulfilling classification criteria for APS. Levels of β_2_GPI in the autoimmune disease controls (without thrombotic events) with persistent aPL did not differ from levels in autoimmune disease controls without aPL, and also were not different from levels in healthy controls.

Subgroup analysis of the total level of β_2_GPI within the APS group revealed no differences between those with and those without an additional autoimmune disease. Furthermore, there was no difference between those with arterial thrombosis and those with venous thrombosis (Supplementary Figure 3, http://onlinelibrary.wiley.com/journal/10.1002/(ISSN)1529-0131).

### APS is associated with a greater proportion of β_2_GPI being in an oxidized state

**Figure 3:**
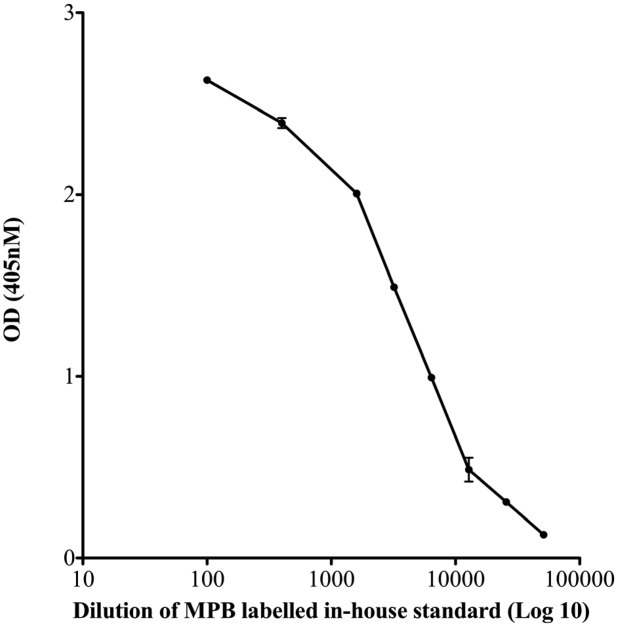
High level of sensitivity of the assay for quantifying relative amounts of reduced β_2_GPI. Pooled human serum from healthy volunteers (n = 10) was labeled with MPB, and a streptavidin-coated plate–based enzyme-linked immunosorbent assay for reduced β_2_GPI was performed on varying dilutions of this labeled sample, as described in Patients and Methods. The linear range for this assay was at dilutions between 1:400 and 1:128,000. OD = optical density (see Figure 1 for other definitions).

Each patient sample was labeled with MPB, and the amount of β_2_GPI in the reduced form was compared and expressed as a percentage of that observed in a pooled standard (derived from 10 healthy volunteers who were matched for age and sex with the APS group), after correction for the total amount of β_2_GPI. The same in-house pooled standard was used for every MPB labeling experiment and assay. The sensitivity for detecting reduced β_2_GPI with this assay extends to a dilution of >128,000-fold, indicating marked sensitivity (Figure 3). The linear range was found to be between dilutions of 400- and 128,000-fold. The dilution found to yield ∼50% of maximum OD was found to be 1:4,000, and hence this dilution was used to screen all patient samples for reduced β_2_GPI. This assay has previously been shown to yield identical results when serum and plasma sampled from the same patient are tested in parallel (13).

**Figure 4:**
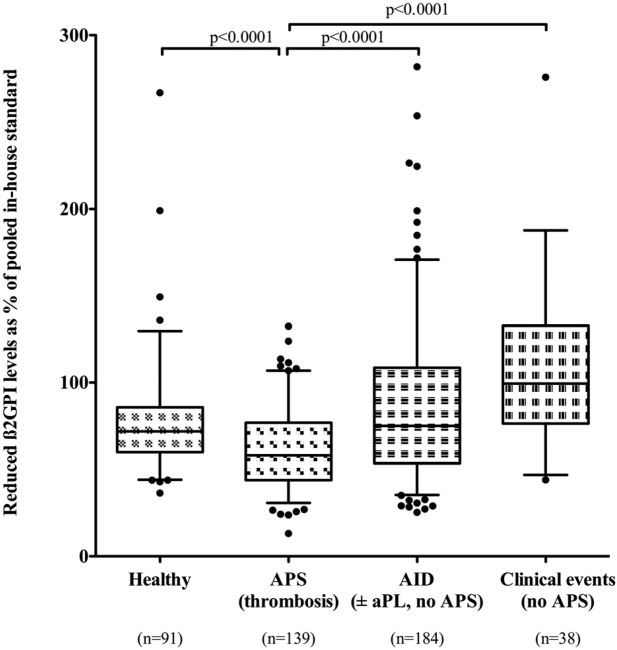
Circulation of β_2_GPI in an oxidized form in patients with APS. Levels of β_2_GPI in the reduced form were assayed and expressed as a percentage of that observed in an in-house standard (pooled serum from 10 healthy volunteers) after correction for the total amount of β_2_GPI. The same pooled standard was used throughout. APS patients presenting with thrombosis had significantly lower amounts of β_2_GPI in the reduced form as compared to each of the 3 control groups. Data are presented as box plots, where the boxes represent the 25th to 75th percentiles, the lines within the boxes represent the median, and the lines outside the boxes represent the 10th and 90th percentiles. Circles indicate outliers. See Figure 2 for definitions.

Figure 4 shows that the relative proportion of β_2_GPI in the reduced form, expressed as a percentage of that observed with the in-house standard, was significantly less in APS patients presenting with vascular thrombosis as compared to healthy controls, autoimmune disease controls, and clinical event controls (all *P* < 0.0001). Thus, β_2_GPI in APS patients presenting with thrombosis is in an oxidized state relative to each of the other 3 control groups. Similar to the findings in the analysis of total β_2_GPI, a lower level of the reduced β_2_GPI (proportion ≤50%) was associated with a greater risk of thrombosis. An OR of 4.1 (95% CI 1.9–8.8) in relation to healthy subjects was observed after adjustment for age and sex. A similar but somewhat smaller effect (OR 2.0 [95% CI 1.2–3.4]) was also obtained when the reference group was patients with autoimmune disease with or without aPL but without thrombosis.

**Figure 5:**
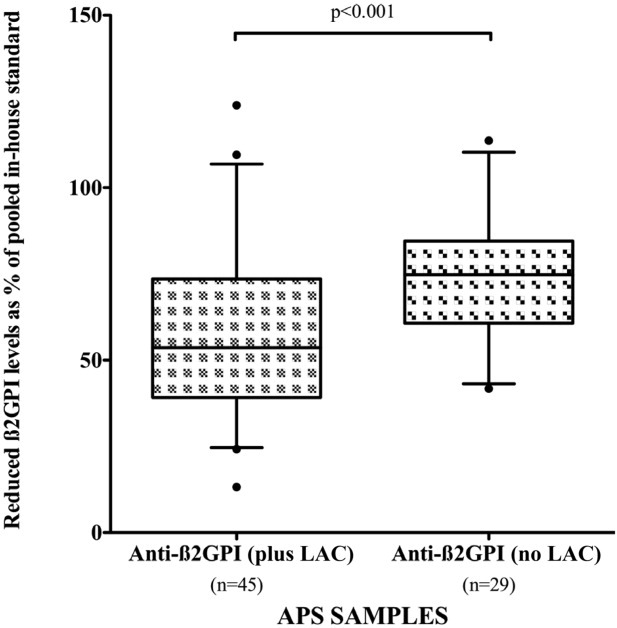
Association of positivity for anti-β_2_GPI combined with lupus anticoagulant (LAC) with an elevated proportion of β_2_GPI circulating in an oxidized state. Samples from APS patients presenting with vascular thrombosis who were positive for both anti-β_2_GPI and LAC had significantly lower amounts of β_2_GPI in the reduced form as compared to those from patients who were positive for anti-β_2_GPI but not for LAC. Data are presented as box plots, where the boxes represent the 25th to 75th percentiles, the lines within the boxes represent the median, and the lines outside the boxes represent the 10th and 90th percentiles. Circles indicate outliers. See Figure 2 for other definitions.

Patient positivity for lupus anticoagulant (LAC) activity has been reported to be a strong predictor of thrombosis compared to anti-β_2_GPI or anticardiolipin antibodies without LAC activity, particularly with regard to arterial thrombosis and the development of stroke (4, 20). Subgroup analysis of the various aPL subtypes within the APS group revealed that the proportion of β_2_GPI circulating in the reduced state was significantly lower in the APS patients who were positive for both anti-β_2_GPI and LAC as compared to those positive for anti-β_2_GPI but not LAC (median 53.58% [IQR 39.18–73.56] [n = 45] versus 74.80% [IQR 60.69–84.51] [n = 29]; *P* ≤ 0.001) (Figure 5). Interestingly, levels of β_2_GPI were also lower in APS patients presenting with arterial thrombosis only (median 53.81% [IQR 39.38–74.62] [n = 67]) versus those presenting with venous thrombosis only (62.09% [IQR 49.64–83.11] [n = 59]) (*P* < 0.045), as shown in Supplementary Figure 4, http://onlinelibrary.wiley.com/journal/10.1002/(ISSN)1529-0131.

## DISCUSSION

This is, to our knowledge, the first reported demonstration that the redox state of the autoantigen β_2_GPI, in conjunction with plasma concentration levels, is different in APS patients compared to healthy or disease control subjects. Our study is the first to definitively confirm that β_2_GPI levels are elevated in APS patients—both those with and those without an additional autoimmune disease—as compared to healthy and disease control groups. The finding of elevated levels of β_2_GPI was observed by our group previously, albeit utilizing far lower numbers of patients (19). In addition, it is reported herein that levels of oxidized β_2_GPI are elevated in APS patients compared to healthy and disease controls. A novel assay to measure relative amounts of reduced β_2_GPI, as well as the ELISA for total β_2_GPI, had good reproducibility and demonstrated strong associations with the APS disease phenotype. The robust nature of these findings is highlighted by the large numbers of well-characterized patients (>450) screened through this large international collaborative multicenter effort coupled with the use of both healthy and 2 distinct disease control groups. Such assays that precisely quantify the amount of posttranslationally modified autoantigen are unique in the field of APS, and even autoimmunity.

An extensive number of in vitro and in vivo studies suggest that anti-β_2_GPI autoantibodies in complex with β_2_GPI directly contribute to the APS clinical phenotype of thrombosis (5). In the present study, we have demonstrated that patients who are persistently positive for aPL and have the clinical features of APS have higher levels of total and oxidized β_2_GPI compared to controls. It is reasonable to hypothesize that clinical states associated with an increased oxidative stress load, such as pregnancy and infection (21), may lead to further increases in the levels of oxidized β_2_GPI in the plasma, potentially elevating the risk of pathologic thrombosis in patients who are positive for anti-β_2_GPI antibodies. This is based on the premise that an increased plasma load of oxidized β_2_GPI may lower the threshold for provoking an anti-β_2_GPI autoantibody–mediated dysregulated prothrombotic response. A recent study demonstrated that oxidative stress may drive β_2_GPI production in vivo through activator protein 1 and NF-κB–mediated up-regulation of β_2_GPI gene promoter activity (22). Hence, an enhanced oxidative stress load may increase antigenic load, potentially driving anti-β_2_GPI production in autoimmunity-prone subjects and lowering the threshold for a clinical event. This hypothesis supports a rationale as to why SLE in particular is associated with anti-β_2_GPI antibodies, given that this condition is characterized by a propensity toward autoreactivity, B cell hyperactivity, and oxidative stress (23, 24).

It was recently shown that β_2_GPI with free thiols protects endothelial cells against oxidative stress–induced cell injury, whereas oxidized β_2_GPI (which lacks free thiols) has no such protective effect (13). Given the present finding that a significant proportion of circulating β_2_GPI is in this protective reduced form in healthy individuals, it may be reasonable to hypothesize that the relative abundance of oxidized β_2_GPI in APS lowers the threshold for development of vascular thrombosis. If this hypothesis is correct, then one would expect elevated levels of oxidized β_2_GPI to represent an independent risk factor for thrombosis. Analysis of posttranslational modifications of β_2_GPI on patient samples collected prospectively and subsequent determination of the presence or absence of a thrombotic event would allow for predictive calculations that could be used to test such a hypothesis.

With the development of novel assays to detect and quantify plasma β_2_GPI–related redox changes, it is expected that stratification of anti-β_2_GPI antibody–positive individuals for thrombotic risk according to the levels of total, reduced, and oxidized β_2_GPI may be possible, with the attendant potential opportunity for implementing medical prophylactic measures during these periods of elevated risk. Prospective longitudinal studies aimed at validating the predictive and diagnostic role of such an approach are needed.
