# Supplementary material for: Novel Assays of Thrombogenic Pathogenicity in the Antiphospholipid Syndrome Based on the Detection of Molecular Oxidative Modification of the Major Autoantigen β2-Glycoprotein I
Source: Arthritis Rheum. 2011 Sep;63(9):2774–82. doi: 10.1002/art.30383 (PMC3328749; doi:10.1002/art.30383)
Supplement: Supplementary file 1 [file art0063-2774-SD1.doc]

**SUPPLEMENTARY INFORMATION**

**Quantification of absolute proportion of ß2GPI with free thiols circulating *in vivo* in healthy volunteers – validation of method.**

Human serum was incubated with the biotin labelled MPB (dissolved in DMF buffer) or DMF buffer alone. Following acetone precipitation to remove free MPB, the labelled and unlabelled serum samples were incubated with streptavidin beads to deplete all biotin labelled proteins. The supernatant was assayed to quantify the amount of ß2GPI in both the MPB and non-MPB labelled samples post streptavidin bead incubation. The relative decrease in OD of the MPB labelled samples thus represents the amount of ß2GPI that may be labelled with MPB, and hence the minimum amount in the serum that has free thiols. The results for this analysis are shown in figure 1. However, prior to performing this experiment the validity of this assay needed to be confirmed by demonstrating three essential requirements:

1. The amount of MPB employed to label serum was the amount that resulted in maximum labelling of ß2GPI
2. No evidence of non-specific (background) binding of unlabelled ß2GPI to streptavidin beads is observed
3. Incubation with streptavidin beads using the method employed indeed depletes the vast majority of MPB labelled ß2GPI.

To answer point i), serum derived from a healthy volunteer (male, age 38 years) was labelled with increasing concentrations of MPB. For each dilution, MPB labelled proteins were then depleted with streptavidin bead incubation and a ß2GPI assay performed. Figure S1*A* shows that there is a linear dose response effect which plateaus at 7.5 mM MPB. Hence the concentration of MPB used to label the pooled serum sample was 9 mM, to ensure saturation of the system. To confirm that ß2GPI did not non-specifically bind to streptavidin beads, a ß2GPI ELISA assay was performed on unlabelled human serum pre and post incubation with streptavidin beads. Figure S1*B* shows that the total amount of ß2GPI does not change following incubation with streptavidin beads. Finally, in order to confirm that incubation with streptavidin beads indeed depletes the vast majority of MPB labelled ß2GPI, MPB labelled serum proteins pre and post biotin depletion were captured by incubation with a streptavidin plate, which was then probed with an anti-ß2GPI antibody. Figure S1*C* shows that 84.3% ± 18.45 (mean ± SD, n=2, p≤0.02) of MPB labelled ß2GPI is depleted by this method.

**Assay for quantifying total ß2GPI levels – specificity and establishment of linear range.**

The assay employed was based on a previously published sandwich ELISA method utilising an in-house purified rabbit polyclonal anti-ß2GPI antibody in the solid phase to capture ß2GPI and a monoclonal murine anti-ß2GPI antibody to detect it. Figure S2 shows that employing these two antibodies in this assay yields a standard curve with a good linear range (between 170 ng/ml to 5 ng/ml). The signal falls to undetectable levels when either a murine isotype control antibody or rabbit polyclonal IgG is employed as a negative control.

**Supplementary Figure legends**

Fig. S1. Optimisation of method for quantifying amount of reduced ß2GPI in human serum. (*A*) Human serum was labelled with increasing concentrations of MPB. MPB labelled proteins were then depleted by incubation with streptavdin beads as described in SI Methods and a total ß2GPI assay performed on each sample. Results are expressed as a percentage of total ß2GPI observed in an unlabelled serum sample post incubation with streptavidin beads. (*B*) A total ß2GPI assay was performed on a human serum sample incubated pre and post biotin depletion with streptavidin beads (*C*) Incubation of biotin labelled ß2GPI with streptavidin beads as described in Methods depletes MPB labelled ß2GPI by 84.3% ± 18.45 (mean ± SD, n=2, p≤0.02 – two-tailed unpaired t-test).

Fig. S2. Assay for quantification of total ß2GPI. Human serum derived from 10 healthy volunteers (age and sex matched with the APS group) was determined to have a total ß2GPI concentration of 170 µg/ml by employing a commercial total ß2GPI ELISA kit (Hyphen BioMed, Neuville-sur-Oise, France). This sample was then used in the in-house total ß2GPI assay utilizing a polyclonal rabbit anti-ß2GPI antibody (10 nM) to capture ß2GPI and a murine monoclonal anti-ß2GPI antibody (25 nM) to detect it. Linearity within this assay is achieved between dilutions 1000 (170 ng/ml of ß2GPI) and 32,000 (5.3 ng/ml). Negligible binding is observed when the plate is coated with (□) normal polyclonal rabbit IgG (10 nM) as a control capture antibody or when a (▲) murine isotype control detection antibody is employed.

Fig S3. Sub-group analysis of total ß2GPI levels in APS. Comparing levels of total ß2GPI in thrombosis associated APS between A) those with and without an additional AID and between B) those with arterial thrombosis (AT) versus those with venous thrombosis (VT).

Fig S4. Sub-group analysis of reduced ß2GPI in APS. Comparing the proportion of ß2GPI in the reduced form in thrombosis associated APS between A) those with and without an additional AID and between B) those with arterial thrombosis (AT) versus those with venous thrombosis (VT).

**Figures**

***A* *B***


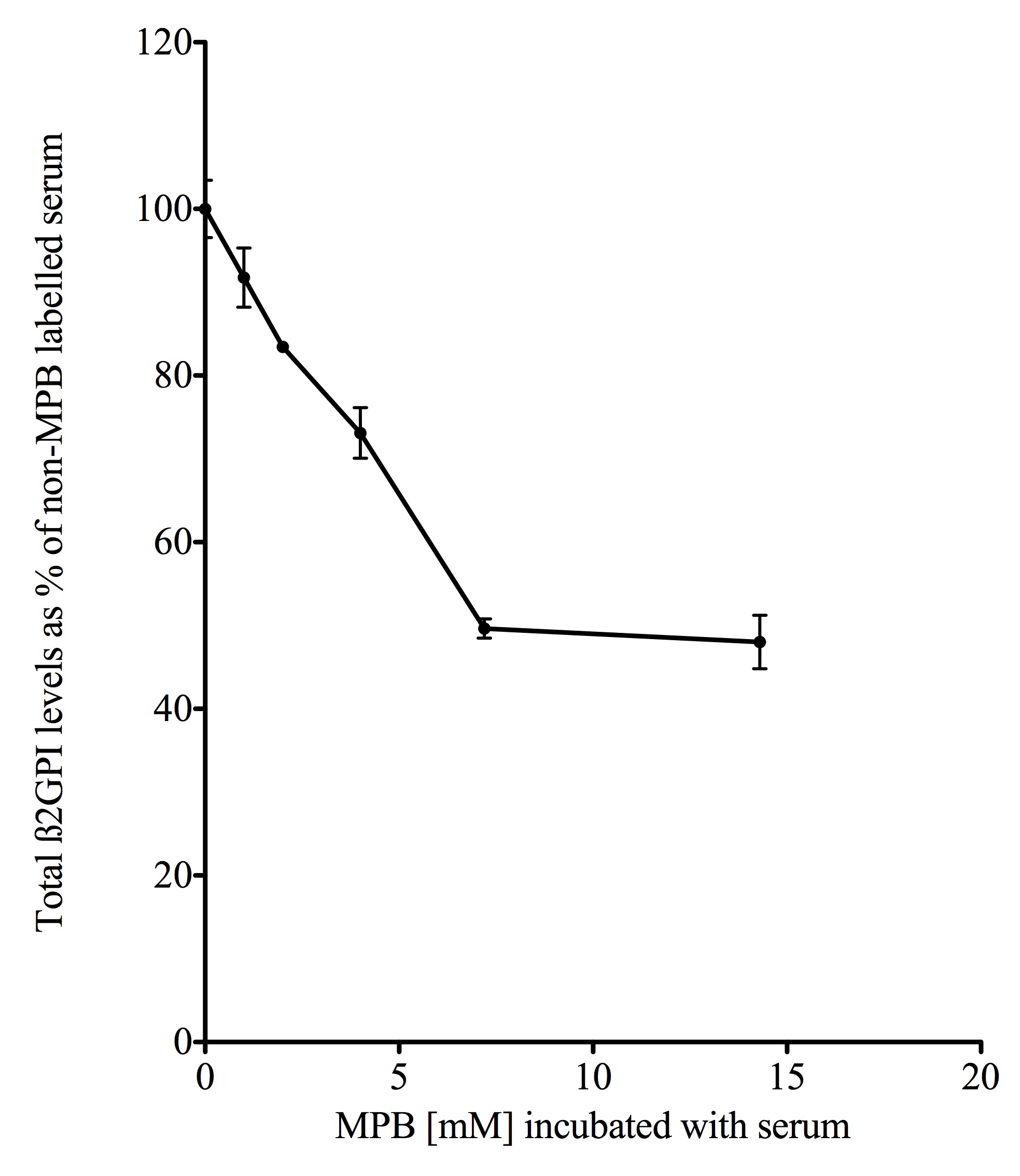

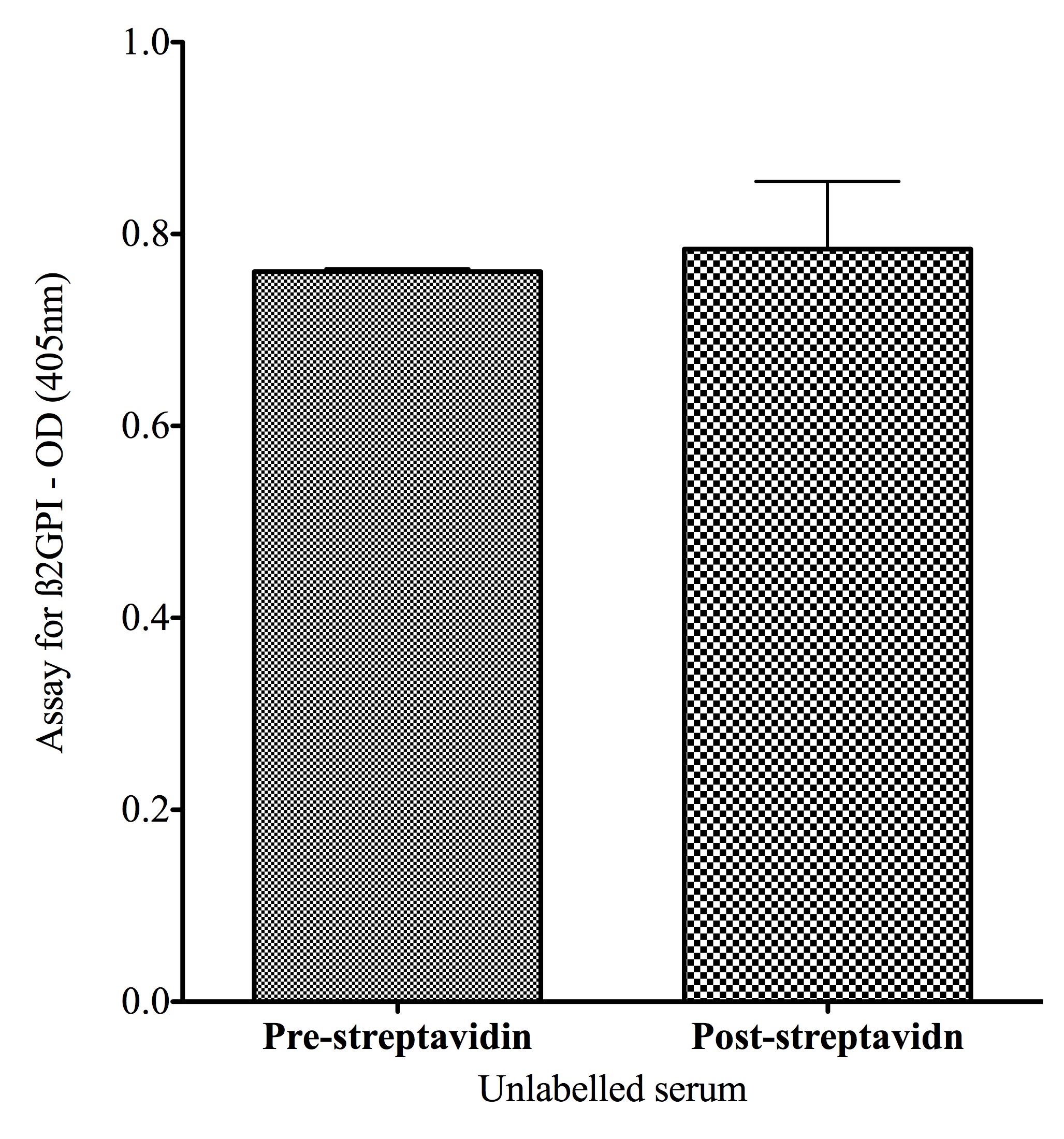


***C***


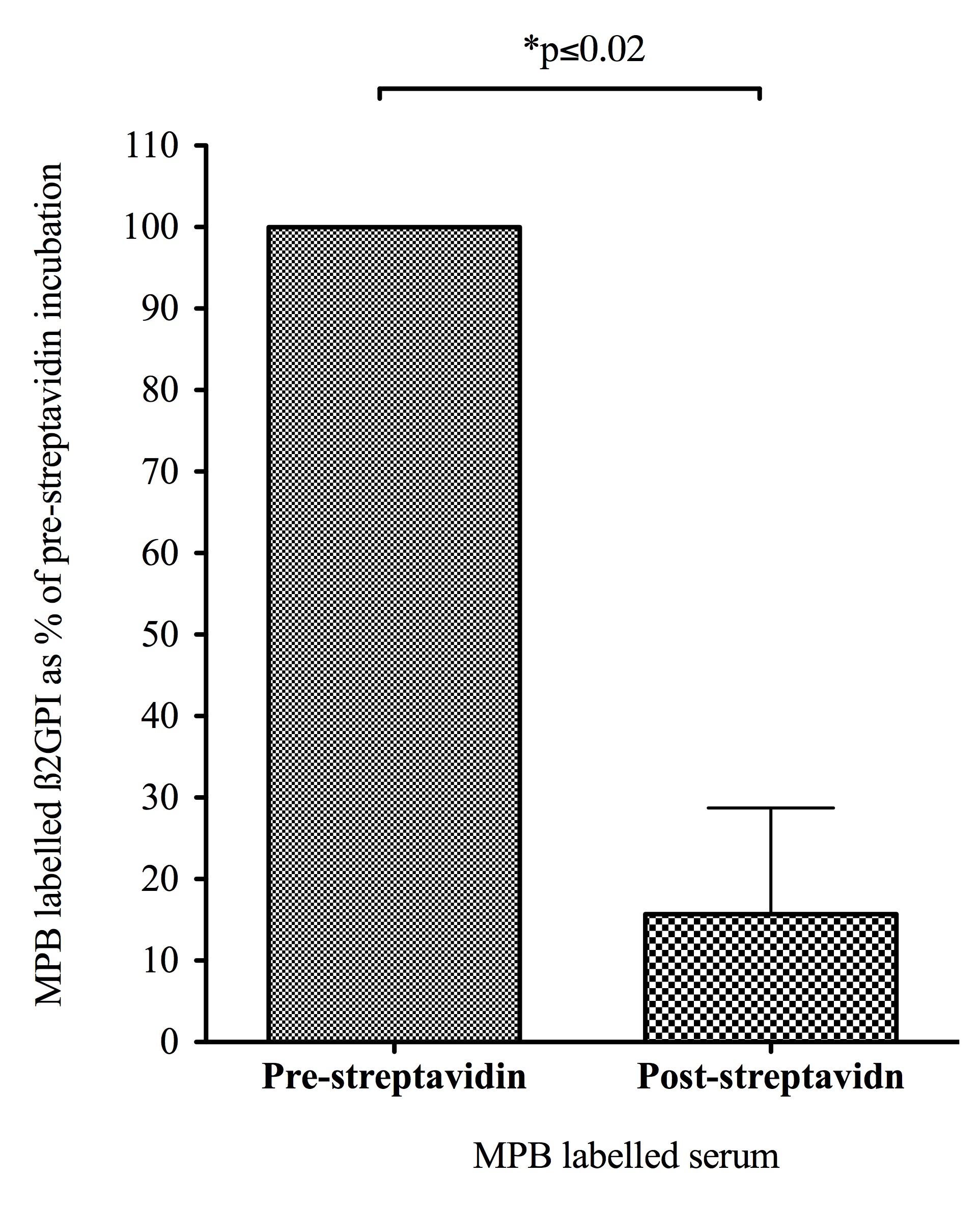


**Figure S1. Optimisation of method for quantifying amount of reduced ß2GPI in human serum**

**
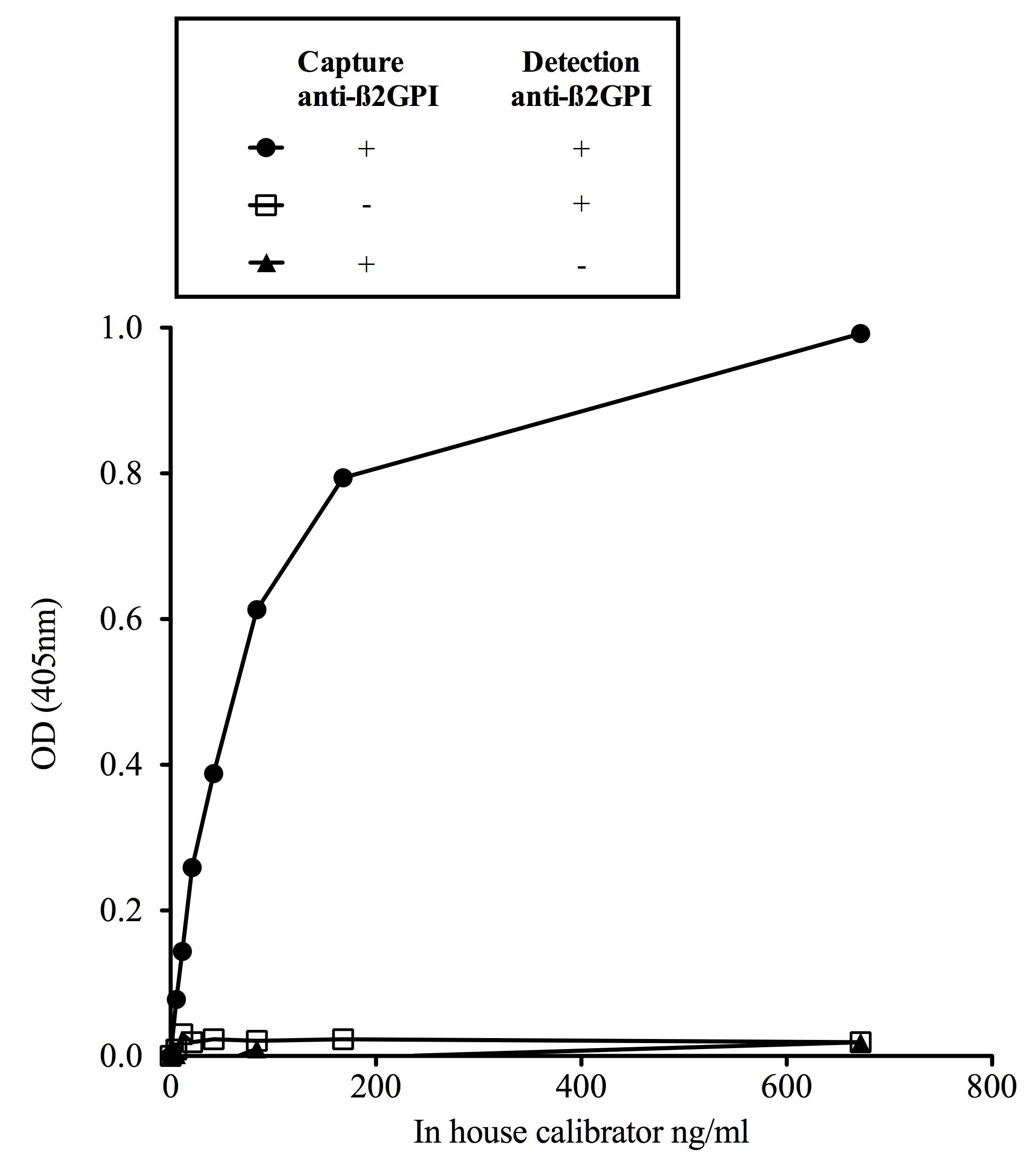
**

**Figure. S2. Assay for quantification of total ß2GPI**

**A
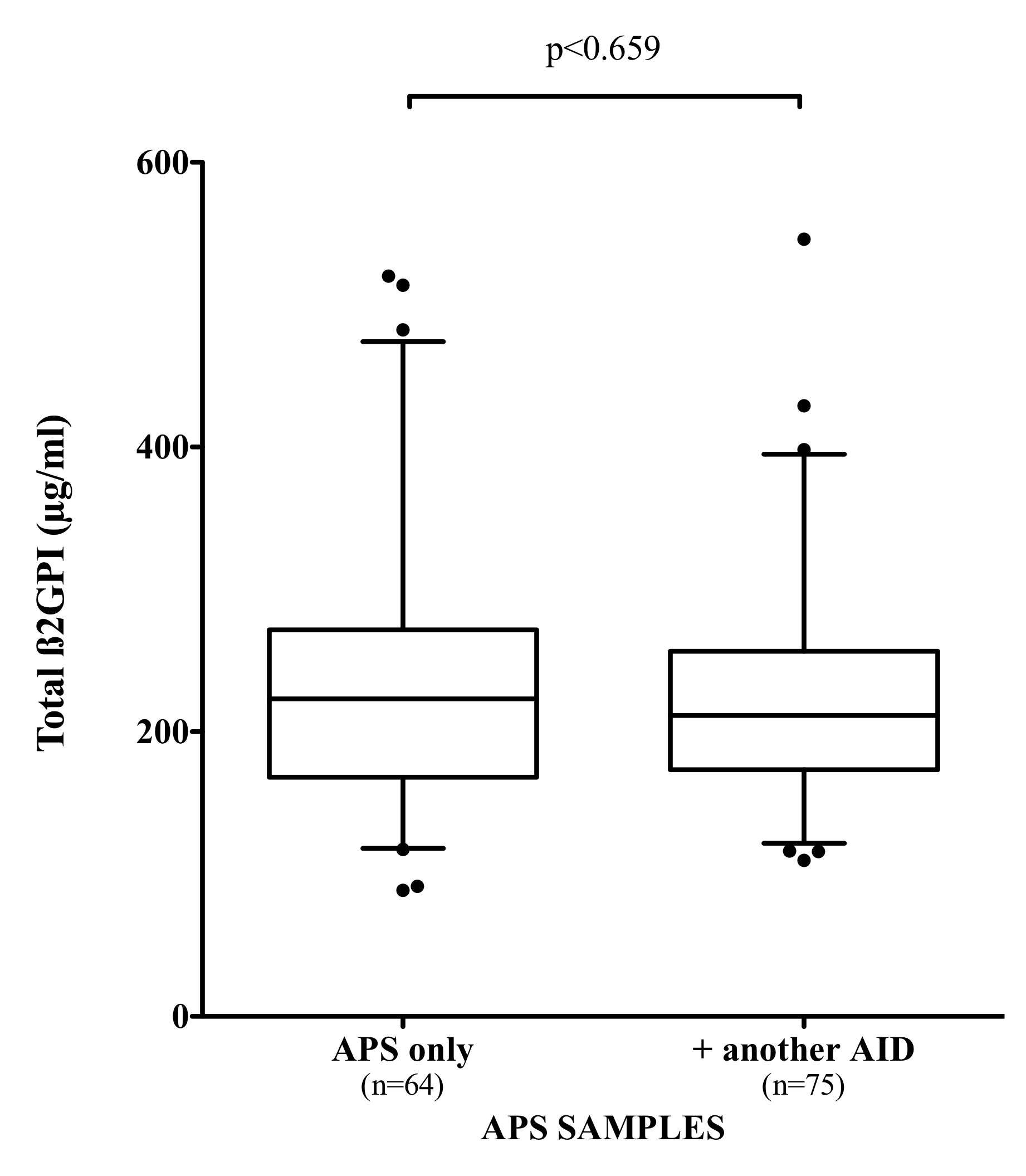
B
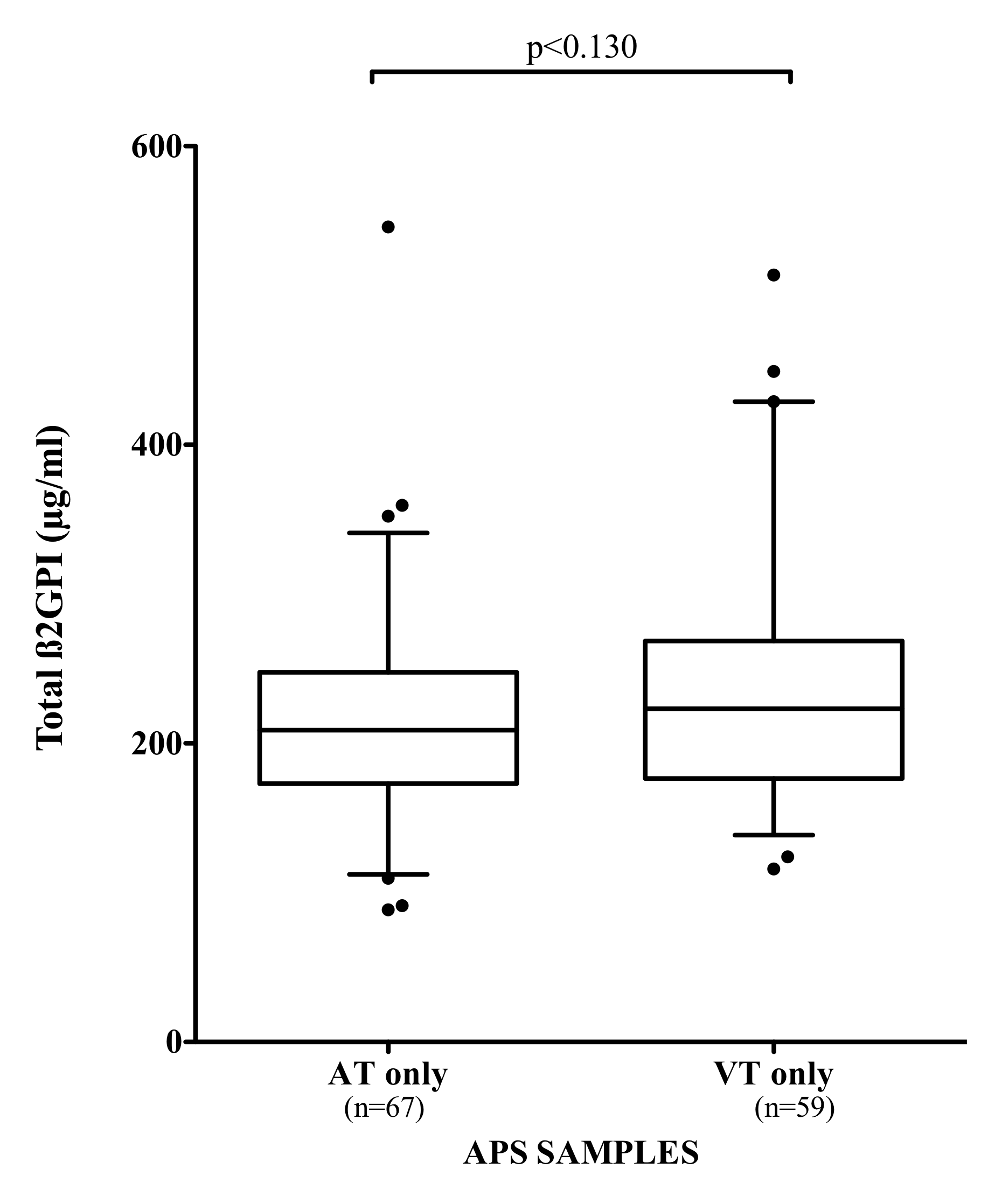
**

**Figure S3. Sub-group analysis of total ß2GPI levels in APS.**

**A
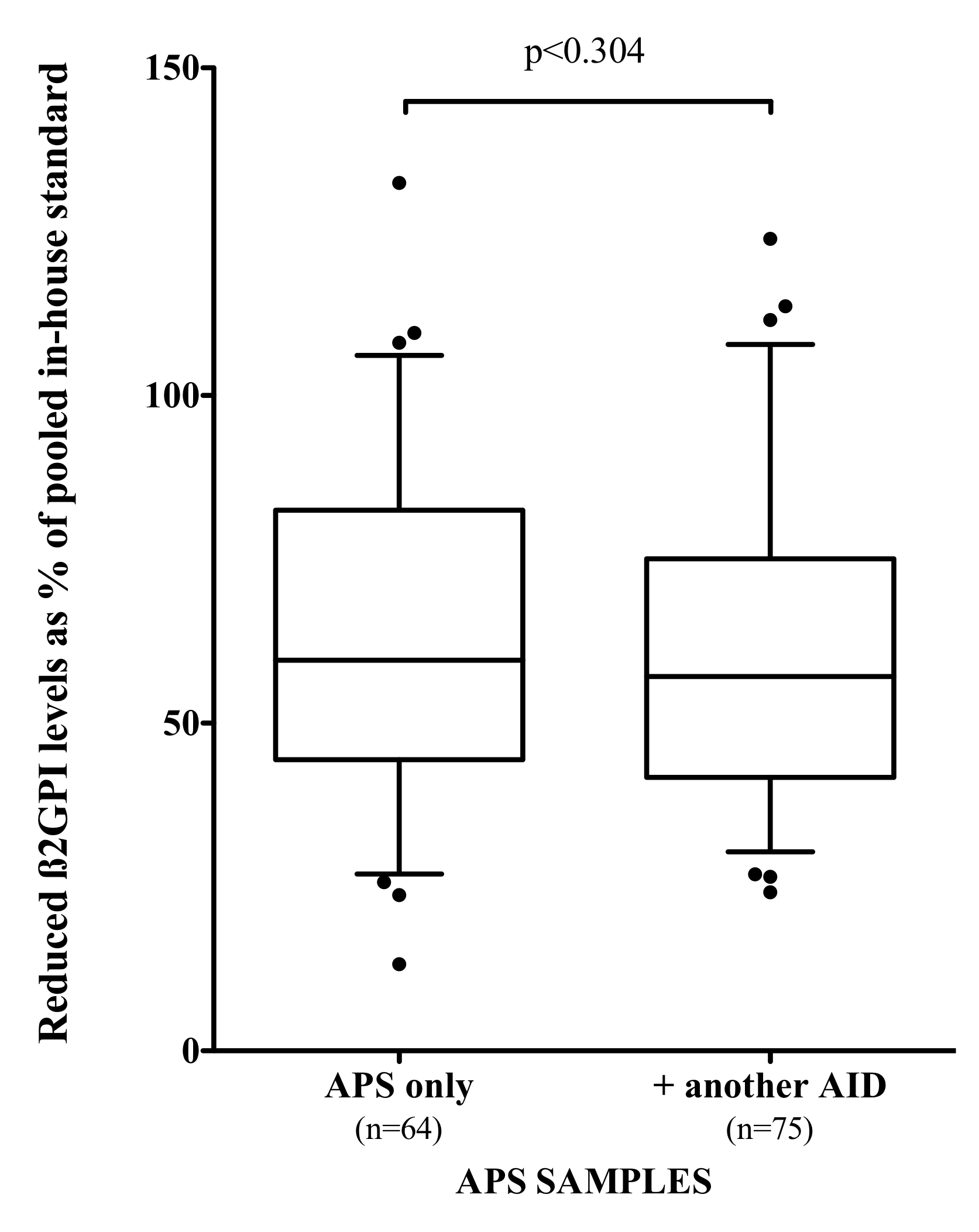
 B
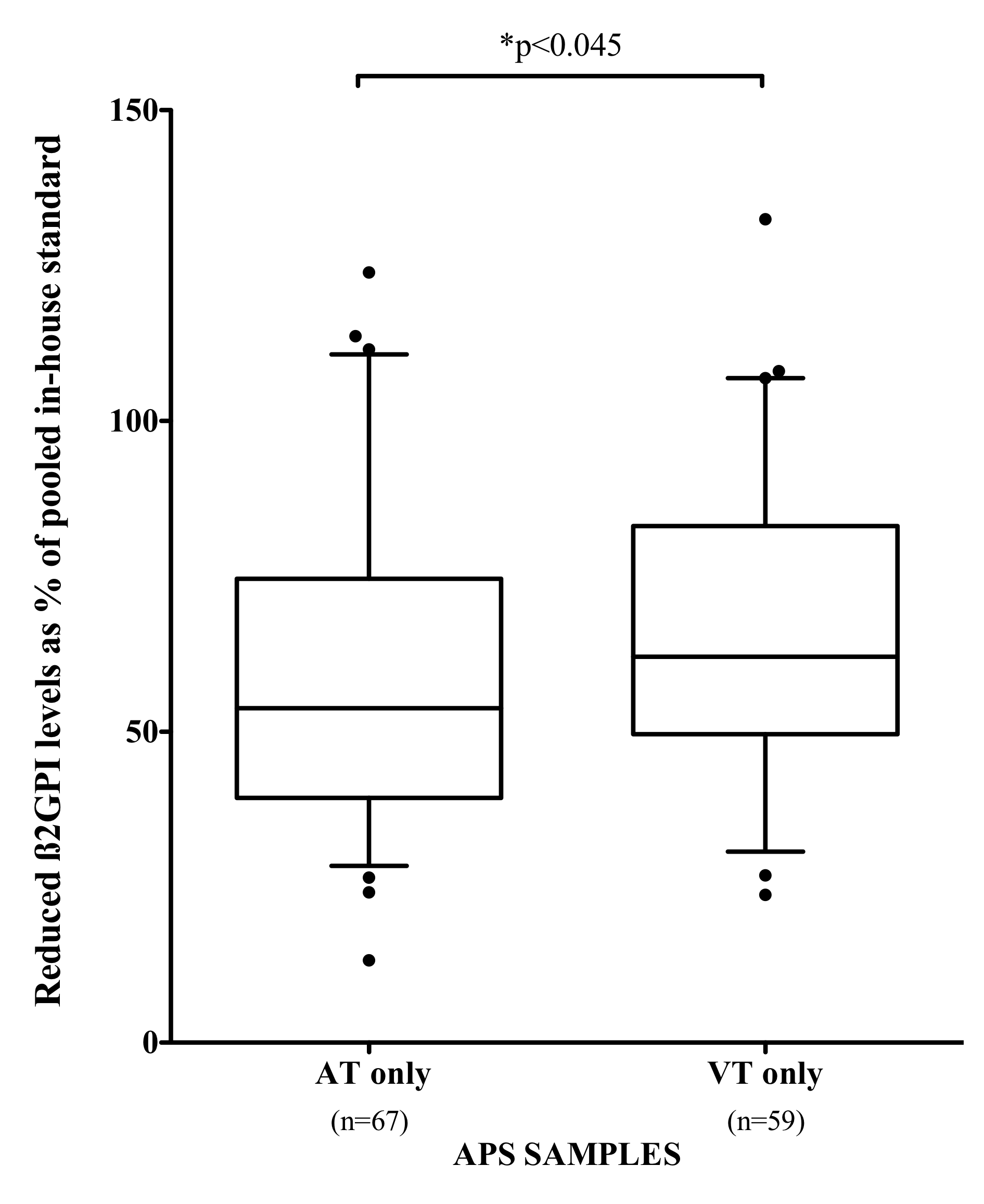
**

**Figure S4. Sub-group analysis of reduced ß2GPI in APS.**
